# Prevention of Venous Thrombotic Events in Brain Injury: Review of Current Practices

**DOI:** 10.5041/RMMJ.10101

**Published:** 2013-01-30

**Authors:** Stuart Glassner, Karan Srivastava, Paul Cofnas, Brian Deegan, Peter DeMaria, Rimsky Denis, Enrique Ginzburg

**Affiliations:** Jackson Memorial Hospital and University of Miami, Florida, USA

**Keywords:** Deep venous thrombosis, prophylaxis, traumatic brain injury, venous thromboembolic events

## Abstract

Venous thromboembolic event after traumatic brain injury represents a unique clinical challenge. Physicians must balance appropriate timing of chemoprophylaxis with risk of increased cerebral hemorrhage. Despite an increase in the literature since the 1990s, there are clear disparities in treatment strategies. This review discusses the prominent studies and subsequent findings regarding the topic with an attempt to establish recommendations using the existing evidence-based literature.

## Introduction

Despite multiple studies investigating prevention of venous thromboembolic events (VTE) with pharmacologic or mechanical strategies for brain trauma patients, there still exists much variability in actual practice. The discrepancy arises in the recognition of intracranial bleeding risk and thereby the appropriate timing of intervention. Due to a paucity of randomized control trials, The Brain Trauma Foundation (2007) offers only level III evidence as expert consensus in recommending the use of VTE prophylaxis with low-dose heparin (unfractionated heparin) or low-molecular-weight heparin (LMWH) in patients with severe traumatic brain injury (TBI).^1^ They give caution due to risk of hemorrhagic expansion but no guideline regarding initiation of treatment or agent selection. This review discusses the prominent studies and subsequent findings regarding the topic, with an attempt to establish recommendations using the existing evidence-based literature.

## INVESTIGATIONAL STUDIES

The body of related literature provides several assumptions followed in this paper for consistency. First are the standard anatomical location definitions for deep venous thrombosis (DVT) and proximal DVT that risk propagation to a pulmonary embolism (PE) which may necessitate a more aggressive intervention. The second accepted assumption is based on the fact that most studies cited below include routine use of mechanical prophylaxis alone or in combination with drug prophylaxis. Prior investigations, most recently by Ekeh et al.,^2^ concluded that physical compression by itself is inadequate to prevent DVT. Finally, standard screening methods for VTE include ultrasound venous Doppler unless noted otherwise, although venogram was the gold standard for diagnosis in early research studies.

VTE as consequence of trauma was formally quantified in 1994 by Geerts et al. in an extensive study published in the *New England Journal of Medicine*.^3^ This prospective study had inclusion of only severe trauma victims (using a cut-off of > 9 on the Injury Severity Score) and did not utilize any VTE prophylaxis during patient course. Instead, a predominance of the 349 patient sample underwent contrast venography 1–3 weeks after admission. The authors found an overall incidence of 57.6% (95% CI 52%–62.8%) incurring DVT and 18.1% with proximal DVT, despite only 1.5% of patients displaying clinical symptoms.^3^ Further stratification of injury based on major location (head versus spine versus lower limbs versus face/chest/abdomen) and type yielded a framework for epidemiologic difference in risks. Subgroup analysis revealed that of 91 patients with head trauma alone or with other injury, 53.8% presented with distal deep vein clots and 19.8% presented with proximal (thigh) deep vein clots, for a total of 73% of this population. It is important to note that of the 51 patients with head trauma as their only injury, the incidence of DVT was 39%. As expected, head trauma with lower extremity trauma experienced a DVT rate of 77%, and the study found femur or tibial fractures were an independent risk factor, along with spinal cord injury, age, surgery, and blood transfusion.^3^ Head injuries were grouped as a whole and not further divided.

TBI-induced coagulopathy contributes risk to this population. A review by Laroche et al. from June 2012 characterizes the phenomenon as a combination of both hypercoagulable and hypocoagulable states.^4^ It is hypothesized that the bleeding diathesis is rooted in the elevated release of brain and systemic tissue factor (TF) due to injury. The resulting over-stimulation of the coagulation cascade can then foster a consumptive coagulopathy, though other mechanisms are being investigated.^4^ Additionally, in 2007 Nekludov et al. further found evidence of TF-triggered hyperfibrinolysis as well as a hypercoagulability with generation of micro-thrombosis.^5^ In a related review of TBI patients, evidence of hemorrhagic progression or new development of ischemic lesions after initial emergency room presentation was found in 85% of patients with laboratory evidence of coagulopathy on admission, as compared to 31% of those with normal levels.^5^ Thus in a variety of analyses, diminished platelet counts, increased partial thrombin time (PTT), and elevated prothrombin time are shown to be predictive markers of mortality in severe TBI. Prothrombin time abnormality was specifically noted to be an independent predictor in the landmark multinational IMPACT study (International Mission for Prognosis and Analysis of Clinical Trials in TBI, IMPACT, 2007).^5^,^6^

However, due to small sample size, Geerts’ earliest paper was not able to state whether brain injury itself is an independent risk factor for thromboembolic events.^3^ Consequently, a flurry of investigational studies appeared in the past few years addressing the question. Most notable was a retrospective study of 2,000 patients by Reiff et al. in 2009.^7^ It found a 3–4-fold increase in DVT incidence among TBI patients stratified by “time to initiation” of either low-molecular-weight heparin or low-dose unfractionated heparin. The groups were all on mechanical prophylaxis and varied from 0 ≤ 24 h, 24–48 h, >48 h, or no intervention given. There was an increased risk throughout the categories, with an absolute risk of 3.6% in the earliest administration group increasing to 15.4% at >48 h. Thus, the study asserts that regardless of timing or use of pharmacologic prophylaxis, TBI counted as a risk but was seen with greater incidence associated with delayed onset of prophylaxis.^7^

The Reiff group further consolidated earlier findings, including that of Denson (2008)^8^ and Nathens (2007)^9^ among others who strongly hinted at a correlation between VTE and head injury. Denson et al. reported a rate of 25% in his study.^8^ Furthermore, in 2010 Ekeh et al. analyzed DVT and pulmonary embolism (PE) rates in 677 TBI patients, comparing incidence in isolated head-injured patients with those who had brain trauma combined with extra-cranial damage.^2^ Similar to Geerts, no medical prophylaxis was given, and patients had scheduled screenings for DVT, with Doppler ultrasound. Each patient received compression devices when two lower extremities were viable. The results supported a strong association between VTE and brain trauma. Additionally, both DVT and PE rates were higher with multiple injuries.

In 2009 Kim et al. published an article in *Neurocritical Care* and found a subtle difference in rates of VTE among patients with subarachnoid hemorrhage, intracerebral hemorrhage, and traumatic brain injury (TBI) with cerebral contusions.^10^ Of the 1,195 patients that met study criteria for inclusion, the incidence of symptomatic VTE for subarachnoid hemorrhage patients, intracerebral hemorrhage patients, and the TBI patients were 6.7%, 2.9%, and 3.8%, respectively, resulting in no significant difference.^10^ As in most studies, severity of injury was not an examined variable. The Scales group indirectly investigated the risk of bleeding in various degrees of intracranial hemorrhage using a decision-point model for medical prophylactic use after 24 hours.^11^ They found that no difference surfaced in rates of DVT regardless of severity in intracranial hemorrhage up to the first 24-hour time-frame, though it is conceivable from earlier studies that this would change as the number of hospital days increased.

The crux of the issue, however, involves the timing of prophylactic intervention. This continues as a provocative issue in the preventative treatment of VTE. It is not uncommon for significant variation to occur even in the same institution as illustrated by an earlier study by Scales et al.^12^ Their 2009 survey questioned 160 Canadian neurocritical care physicians (some at the same hospital) and found anything but a consensus on initiation of pharmaceutical prophylaxis. This followed Carlile in 2006 who published the results of an intensive multicenter survey of US practice patterns involving VTE screening and prophylaxis after TBI in the acute care setting.^13^ The data suggested inconsistent use of drug prophylaxis (56%) with more common utilization of mechanical device (69%) in these patients. This lack of consensus echoes findings in other reports that mention a scenario akin to “attending-based medicine” whereby use and timing of chemoprophylaxis is subject to physician or surgeon discretion.

A recent *Journal of NeuroTrauma* article by Dudley et al. may offer insight into the debate.^14^ The study looked at a broader scope of TBI patients and used serial CT scans as a marker of intracerebral hemorrhage stability prior to giving LMWH if no confounding coagulopathy. They chose administration at 48–72 hours, citing prior data that withholding prophylaxis for more than 4 days tripled VTE risk.^7^,^9^,^15^ The population included a spectrum of patients with moderate to severe brain injuries, Glasgow Coma Score varying from 3 to 12, and Injury Severity Score ranging from 4 to 66. Their results showed overall VTE incidence at 7.3% with one death resulting from hemorrhagic expansion as revealed by a follow-up CT scan.

It is duly noted that this study had higher rates of VTE than those intervening at 24 hours, which in fact is what the Reiff study (see above) illustrated with its treatment groups receiving prophylaxis at <24 h, 24–48 h, and >48 h.^7^ Both papers infer that delays of even 24 hours can contribute to VTE risk.^6^,^14^ However, this certainly must be balanced with risk of intracerebral hemorrhage expansion, resulting in a risk-to-benefit ratio directing chemoprophylaxis initiation at the 48–72-hour time-frame.

The Dudley study was the first to compare common LMWH agents, enoxaparin and dalteparin, directed by the prior findings by Geerts (1996) who showed a superiority of enoxaparin to unfractionated heparin.^14^ In the 267-patient retrospective study, the Dudley team found essentially no difference between either LMWH agent in preventing VTE. The investigation did initially reveal a small difference in risk between the two agents; however, the authors cite a negligible discrepancy once baseline characteristics, such as lower starting Glasgow Coma Score in the dalteparin intervention group, were considered. A related 42-month cohort analysis by Minshall et al. in 2011 compared outcome in 386 patients based on type of medical prophylaxis given, but a firm time to initiation of therapy was not delineated.^15^ It inferred patients receiving unfractionated heparin had an increased rate of PE (3.7%) against those receiving LMWH (0%; *P* < 0.05). No hemorrhagic complications occurred in either group. However, the conclusions of this analysis were very limited given that patients with less severe injuries mostly received LMWH, while those with more severe injury were treated with unfractionated heparin. Furthermore, the study had no routine DVT or CT screening and relied solely on clinical judgment versus imaging.^15^

The objection from clinicians about early use of LMWH or unfractionated heparin, within 24 hours, is controversial due to concerns of hemorrhagic extension. However, some reports are worth considering, including those of Norwood et al.^16^,^17^ Altogether 177 patients were included in this study of whom 150 received enoxaparin at 24 hours after admission for intracerebral hemorrhage or after craniotomy for the injury. CT screening was performed at various intervals including admission, 24 hours after hospitalization, and then after receiving the LMWH. After administration of chemoprophylaxis, 4% of patients developed further hemorrhage as evidenced on CT head scans.

The study raised the question whether hemorrhagic changes on CT scan are a consequence of the prophylaxis or a natural progression of the TBI. The analysis did not have a control group.^16^,^17^

## CONCLUSION

Although trauma is a well-established etiology for thromboembolic events, only in the past decade have TBI patients been recognized with an increased risk for VTE. This is most likely due to lengthy hospital immobilization combined with delays in VTE drug regimen prophylaxis. Additionally, the native levels of TF already residing in the brain increase during injury, along with circulating inflammatory cytokines which favor systemic hypercoagulation. Unfortunately, no significant randomized control trials exist with large patient populations, leaving clinicians with expert consensus and a series of retrospective or prospective studies. At issue is the balance between hemorrhagic extension and VTE risk. These important studies yield information about the highest-risk individuals with inference toward practice parameters. Following these, clinical assessment might appear as follows:

- Establish degree of TBI severity and carry out a survey of additional injuries, especially of the lower extremities.
- Start patient on mechanical compression prophylaxis within 24 hours of admission unless contraindicated.
- As mechanical intervention is insufficient alone, LMWH or unfractionated heparin should be started, if no confounding coagulopathy. Timing of this step is variable and controversial. However, a comfortable balance point between extension of the bleed and VTE risk appears to be 48–72 hours. Again, this is inferred from leading studies and not level I evidence. This differs from the Brain Trauma Foundation which omits timing guidelines. Several studies utilized serial head CTs for bleeding progression both before and after anticoagulant administration.
- The choice of regimen, unfractionated heparin versus LMWH, appears only mildly significant in the brain trauma patient. A number of studies show a superiority of LMWH, but bleeding risks are still a concern < 24 hours of initiating therapy. Options within LMWH indicate no superiority between enoxaparin or dalteparin, though the q12 hour dosing of the former may prove of greater benefit than daily dosing of dalteparin.
- The predominance of institutions utilized ultrasound venous Doppler in addition to clinical acumen.

In summary, the evidence above has directed the American College of Chest Physicians to recommend the following (2012)^18^:

- For major trauma patients at high risk for VTE (including brain trauma): mechanical prophylaxis in addition to pharmacologic prophylaxis when not contraindicated by lower-extremity injury.
- For major trauma patients: Use of LMWH or unfractionated heparin or mechanical prophylaxis indicated over no prophylaxis.
- For major trauma patients in whom LMWH and unfractionated heparin are contraindicated: Use of mechanical prophylaxis indicated over no mechanical prophylaxis.
- For major trauma patients: Use of inferior vena cava filter for primary VTE prevention not indicated.

## Abbreviations:

DVT: deep venous thrombosis
GCS: Glasgow coma score
ICH: intracerebral hemorrhage
LMWH: low-molecular-weight heparin
PE: pulmonary embolism
PT: prothrombin time
PTT: partial thrombin time
SAH: subarachnoid hemorrhage
TBI: traumatic brain injury
TF: tissue factor
UFH: unfractionated heparin
VTE: venous thromboembolic events.
